# A Conformationally Constrained Cyclic Acyldepsipeptide Is Highly Effective in Mice Infected with Methicillin-Susceptible and -Resistant *Staphylococcus aureus*

**DOI:** 10.1371/journal.pone.0153912

**Published:** 2016-04-21

**Authors:** Marios Arvanitis, Gang Li, De-Dong Li, Daniel Cotnoir, Lisa Ganley-Leal, Daniel W. Carney, Jason K. Sello, Eleftherios Mylonakis

**Affiliations:** 1 Infectious Diseases Division, Rhode Island Hospital, Providence, RI, United States of America; 2 Warren Alpert Medical School of Brown University, Providence, RI, United States of America; 3 Department of Pediatrics, Hasbro Children's Hospital, Brown University Alpert Medical School, Providence, RI, United States of America; 4 Department of Chemistry, Brown University, 324 Brook Street, Providence, RI, United States of America; ContraFect Corporation, UNITED STATES

## Abstract

**Background:**

Cyclic acyldepsipeptides (ADEPs) are a novel class of antibacterial agents, some of which (*e*.*g*., ADEP 4) are highly active against Gram-positive bacteria. The focus of these *in vivo* studies is ADEP B315, a rationally designed compound that has the most potent *in vitro* activity of any ADEP analog reported to date.

**Methods:**

*In vivo* efficacy experiments were performed using lethal intraperitoneal mice infection models with a methicillin-sensitive *S*. *aureus* (MSSA) and a methicillin-resistant (MRSA) strain. The infected mice were treated with ADEP B315, a *des*-methyl analog of ADEP 4, vancomycin, or the vehicle used for the ADEPs and their survival was assessed daily. A subset of MSSA-infected mice was sacrificed soon after inoculation and the bacterial burden was measured in their livers and spleens. The toxicity of ADEP B315 was assessed in viability assays using human whole blood cultures.

**Results:**

In the MSSA experiments, all mice treated with the vehicle succumbed to the infection within 24 hours. All tested compounds were effective in prolonging survival of infected mice (p<0.001). Mice treated with ADEP B315 had a 39% survival rate by 10 days compared to 7% survival in mice treated with a des-methyl ADEP 4 analog (p = 0.017). Survival of the infected mice treated with ADEP B315 was comparable to those treated with vanocmycin (p = 0.12) at the same dose. Further, bacterial burden in the liver and spleen was significantly lower in mice treated with ADEP B315 compared to controls. In the MRSA experiments, ADEP B315 was able to significantly prolong survival compared to mice treated with either the vehicle (p = 0.001) or vancomycin (p = 0.007). ADEP B315 exhibited no significant toxicity in human whole blood cultures at concentrations up to 25 μg/ml.

**Conclusions:**

ADEP B315 is safe and can cure mice that have lethal infections of methicillin-sensitive and -resistant strains of *S*. *aureus*.

## Introduction

Given the inevitability of antibacterial resistance and the increasing frequencies with which it is observed in clinical isolates of pathogenic bacteria, the development of novel antimicrobial agents is an ongoing necessity. A four decade-long innovation gap with respect to the chemical structures and mechanisms of antimicrobial drugs has contributed to our declining capacity to treat infectious diseases [1]. There is a growing appreciation of the antibacterial resistance crisis and its impact on the treatment of numerous infectious diseases [2, 3]. Clearly, there is an urgent need to discover and develop antimicrobial drugs that have novel structures and/or mechanisms of actions.

Among the most promising antibacterial drug leads to emerge in the past decade are the cyclic acyldespsipeptides (ADEPs) [4]. The prototypical members of this group of antibiotics are “A54556A and B” produced by *Streptomyces hawaiiensis* and enopeptins A and B produced by *Streptomyces* sp. RK-1051 [5, 6]. These molecules have potent activity against Gram-positive bacteria, including drug-resistant pathogens like *Staphylococcus aureus*, *Streptococcus pneumoniae*, and *Enterococcus* spp. [4]. The ADEPs have captured the attention of the scientific and medical communities because their mechanism of action is distinct from all currently marketed antibacterial drugs. The cellular target of the ADEPs is the tetradecameric peptidase called ClpP [4, 7, 8]. Binding of the ADEPs to ClpP induces dramatic changes in its quaternary structure that expose active sites that are otherwise physically sequestered [9, 10]. This conformational change of the peptidase leads to uncontrolled proteolysis and cell death [4, 7, 8].

**Fig 1:**
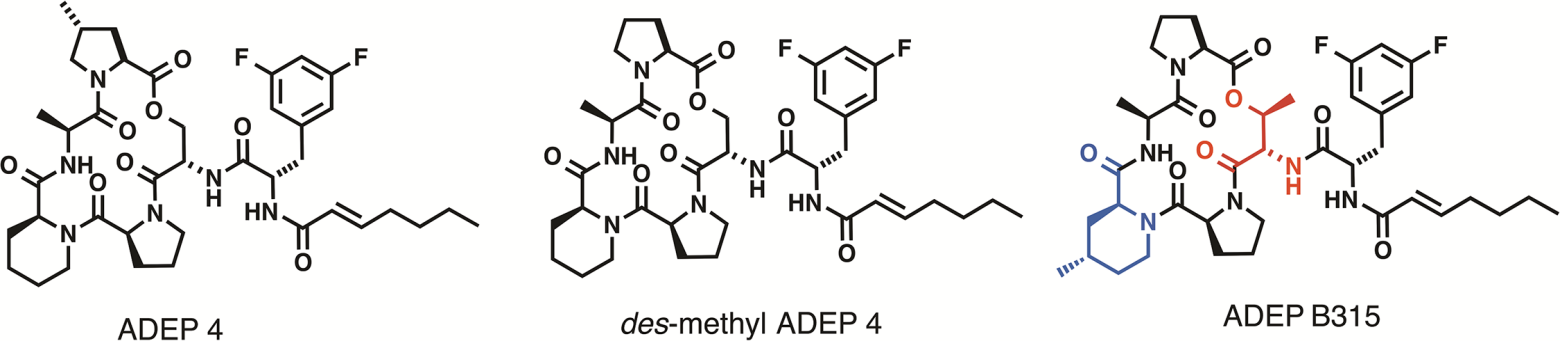
Chemical structures of ADEP 4, *des*-methyl ADEP 4, and ADEP B315. The conformationally constraining amino acids of ADEP B315 are highlighted (4-methyl pipecolic acid in blue and allo-threonine in red).

Despite strong potency in *in vitro* assays, the ADEP natural products have poor efficacy in animal models of infection [4]. Accordingly, the ADEPs have been the subject of medicinal chemistry programs that have yielded analogs with improved pharmacological programs [4, 11–13]. The best characterized of these analogs is called ADEP 4 [4, 11] (Fig 1). This compound has improved activity *in vitro* and is efficacious in animal models of *S*. *aureus* and *S*. *pneumoniae* [4, 11]. ADEP 4 has also been reported to have efficacy against biofilms and to have a sterilizing activity when used in combination with rifampicin [14]. Recently, Sello and co-workers reported rational modifications of the ADEP 4 structure that led to significant improvements in biological activity [13]. They found that substitution of the pipecolate and serine residues of the ADEP 4 peptidolactone with more conformationally constrained analogs (*i*.*e*., 4-methyl pipecolate and *allo*-threonine, respectively) rendered the molecules with rigidity that translated to improved ClpP binding and cell permeability. The most rigid analog of ADEP 4, noted here as ADEP B315, exhibited exceptional activity against *S*. *aureus*, *S*. *pneumoniae*, and *Enterococcus faecalis* [13] (Fig 1). For instance, the MICs of ADEP B315 against *S*. *pneumoniae* and *E*. *faecalis* were as low as 20 picograms/mL [13]. Remarkably, these MICs were up to 200-fold lower than those reported for ADEP 4 [4, 11]. The potency of ADEP B315 in the *in vitro* assays and structural similarity to ADEP 4 suggested that it could be especially efficacious as an antibacterial agent *in vivo*. To define pharmacological potential of ADEP B315, we assessed its toxicity and its efficacy in mouse models of *S*. *aureus* peritonitis. We also compared its efficacywith a bioactive and easily prepared *des*-methyl analog of ADEP 4 (having a proline residue in the peptidolactone rather than a 4-methyl proline) (Fig 1) and with vancomycin.

## Methods

### Survival assays

Evaluations of ADEPs in murine peritonitis models were carried out as previously described [4]. Female 8 week-old CFW mice were used in all experiments. The infected mice were treated with ADEP B315, a *des*-methyl analog of ADEP 4, or vancomycin (Fig 1). The vehicle used for the ADEPs was 75% PEG400/10% Ethanol/15% water, whereas that used for vancomycin was water. Vancomycin and the ADEP vehicle were used as positive and negative controls, respectively. For the experiments with methicillin-sensitive *S*. *aureus* (MSSA), *S*. *aureus* ATCC 29213 was grown overnight in trypticase-soy (TS) broth at 37°C with agitation. The cells were harvested by centrifugation, washed three times, and re-suspended in PBS at a concentration of 10^10^ cfu (colony forming units)/mL. Subsequently, 100 μLof this bacterial suspensionwere injected intraperitoneally intomice that had been dosed at 25 mg/kg via the peritoneum with one of the antibacterial agents or the vehicle in 100 μL volumes 30 minutes prior to bacterial inoculation. Approximately one hour after inoculation, the mice weregiven the same intraperitoneal dose of the antibacterial agent or the vehicle. Eight to ten mice were used per group in each experiment. All mice were evaluated daily for survival. The same experiment was repeated twice.

Of note, we confirmed the stability of the experimental compounds in the vehicle well beyond the time required for the animal experiments (data not shown). Since we did not perform pharmacokinetic/pharmacodynamic evaluation of the new compounds, we did not do *in vitro* assessment of the minimal inhibitory concentrations (MICs) of the tested compounds. However, we should note that the MICs of ADEP B315 and the des-methyl ADEP 4 analog against *S*. *aureus* ATCC 29213 have been previously reported [13].

For the experiments with methicillin-resistant *S*. *aureus* (MRSA), the *S*. *aureus* MW2 strain was grown and prepared for inoculation as described above. Each mouse was given 10^9^cfu of bacteria via an intraperitoneal injection. Unlike the aforementioned experiments with MSSA, the mice infected with MRSA were not pre-treated with the antibacterial agents. They were intraperitoneally dosed with each compound at 25 mg/kg or with the vehicle, 15 minutes and 3 hours after the infection. Eight mice were used per treatment group and their survival was monitored on a daily basis. The same experiment was repeated twice.

For all animal experiments survival was monitored at least twice daily, and moribund animals as indicated by: lethargy, decreased mobility/activity, hunched posture, rough hair coat or porphoryin staining were sacrificed by CO2 inhalation followed by cervical dislocation. There were no unexpected animal deaths. We used the humane endpoint as described above to euthanize the animals in order to minimize suffering and distress.

### Determination of bacterial burden in organs from infected and treated mice

Organs were collected from a subset of the mice used inthe MSSA survival assay. Three hours after administration of the antibacterial agents in each experiment, three mice per treatment group were sacrificed via CO_2_ inhalation and part of their livers and spleens were harvested, weighed, and homogenized by grinding. Serial dilutions of the homogenates were plated on TS agar and incubated overnight at 37°C. The bacterial cfus per gram of tissue were enumerated the following day.

### Assays of compound toxicity in healthy mice

For the toxicity testing, healthy female 8 week-old CFW mice were injected with 50 mg/kg of ADEP B315 or vancomycin in 200μL volumes. Three hours later, the mice were euthanized via CO_2_ inhalation and cervical dislocation. Their kidneys and livers were harvested and incubated in 10% formalin solution overnight. Subsequently, thin sections of the organs were made, stained with hematoxylin and eosin, and visualized microscopically. Two mice were used per group.

### Human whole blood cell assay of compound toxicity

Upon informed consent, 5 mL of peripheral blood was drawn into heparinized tubes from 5 subjects aged 18–50 with no clinical or self-reported evidence of bacterial infection. Whole blood was plated 1:3 in cell culture media containing RPMI, 10% fetal calf serum, 100 U/mL penicillin, 100 U/mL streptomycin, and 2 mM L-glutamine (Fisher Scientific) in a 24-well plate in the presence of increasing concentrations (5, 10, 25, 100, 250 μg/ml)of ADEP B315 or the vehicle (75% PEG400/10% Ethanol/15% water). The cells were cultured for 24 hours and processed for flow cytometry. Briefly, blood cultures were treated with 2 mL of FACS lysis buffer (BD Pharmingen) for 30 minutes at room temperature. Cells were washed with phosphate-buffered saline (PBS) and evaluated for viability by flow cytometry using gates generated with propidium iodide and Annexin V.

### Statistical analyses

Survival was plotted using Kaplan Meier curves and p-values were calculated using the log-rank test. Cfu counts were analyzed using one-way ANOVA with Bonferroni post-test calculation. GraphPad 5.0 was used for all statistical calculations. A p value of <0.05 was considered statistically significant.

### Ethics statement

All animal experiments were approved by the Institutional Animal Care and Use Committee of Rhode Island Hospital and were performed according to standard laboratory protocols. Human whole blood samples for toxicity tests were drawn after written informed consent of the participants, and the blood collection was approved by the Rhode Island Hospital Institutional Review Board.

## Results

### ADEP treatment prolongs survival in MSSA infected mice

**Fig 2:**
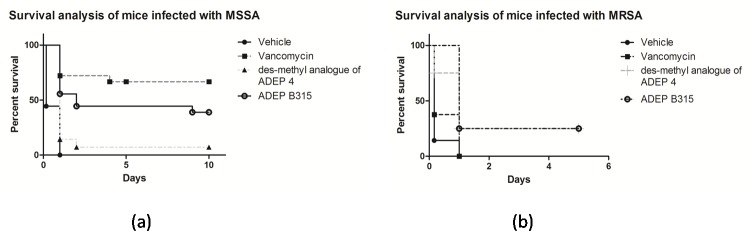
**Kaplan Meier survival curves for MSSA (a) and MRSA (b) infected mice.** All mice were infected with 10^9^cfu of *S*. *aureus* intraperitoneally. For the MSSA assay, each group of mice was pretreated with 25 mg/kg of a compound 30 minutes before inoculation and was also given an additional 25 mg/kg dose 1 hour after inoculation. Control mice were injected twice with the vehicle at the same time-points as treated mice. The survival curves are the combined result of two repeat experiments. Both experiments showed similar findings. For the MRSA murine infection model, each group of mice was treated with two doses of 25 mg/kg of a compound, 15 minutes and 3 hours after inoculation. Control mice were injected twice with the vehicle at the same time-points as treated mice. Eight mice were used per group. Survival was plotted using Kaplan Meier curves and p-values were calculated using the log-rank test.

Results of the MSSA survival experiments are summarized in Fig 2A. All control mice treated with the vehicle alone succumbed to the infection within 24 hours. Infected mice treated with vancomycin or ADEP B315 had 10-day survival rates of 67% and 39%, respectively. On the other hand, only 7% of the infected mice that were treated with *des*-methyl ADEP 4 survived for 10 days. Treatment with the antibacterial agents significantly improved survival as compared to that with the vehicle (p<0.001 for all compounds). Treatment with ADEP B315 resulted in significantly higher survival rates compared to that with *des*-methyl ADEP 4 (p = 0.017). Infected mice treated with vancomycin also had better outcomes than those treated with *des*-methyl ADEP 4 (p<0.001). It is noteworthy that the difference in survival rates of infected mice treated with ADEP B315 and vancomycin was not statistically significant (p = 0.12).

### ADEP treatment prolongs survival in MRSA infected mice

The results of the MRSA survival experiments in mice are summarized in Fig 2B. Two of the eight infected mice in the groups treated with either ADEP B315 or *des*-methyl ADEP 4 survived for five days. In contrast, mice treated with vancomycin or the vehicle died within 24 hours. Treatment with ADEP B315 and *des*-methyl ADEP 4 were able to markedly prolong the survival of infected mice (p = 0.001 and p = 0.02, respectively). Further, ADEP B315 outperformed vancomycin in the mice survival experiments (p = 0.007).

### ADEP treatment reduces bacterial burden in infected mice

**Fig 3:**
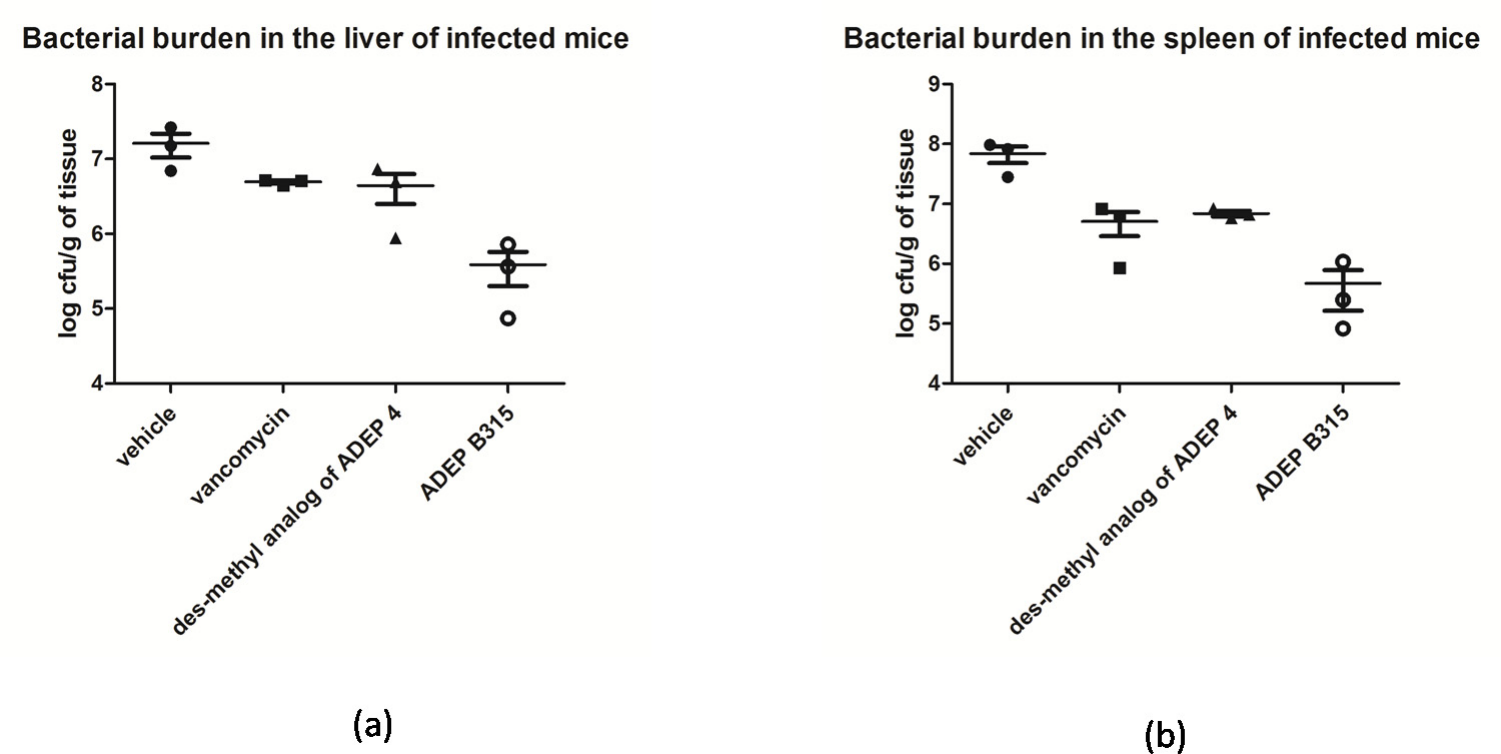
***S*. *aureus* log colony forming units/ gram of tissue in the liver (a) and spleen (b) of infected mice.** All mice were treated at the exact same way as in the MSSA survival assay, with the exception that they were sacrificed via CO_2_ inhalation 3 hours after inoculation and their liver and spleen were collected for cfu determination. Three mice were used in each group. Cfu counts were analyzed using one-way ANOVA with Bonferroni post-test calculation.

The measurements of the bacterial burdens in the livers (Fig 3A) and spleens (Fig 3B) of the MSSA infected mice that were treated with the antibacterial agents or the vehicle were made and are expressed as cfus. Relative to the results of control experiments wherein mice were treated the vehicle, it was apparent that treatment with either the ADEPs or vancomycin significantly reduced the number of *S*. *aureus* cfus in the spleens of infected mice (p<0.01 for all compounds). On the other hand, only treatments with the ADEPs significantly reduced the bacterial burdens in the livers of infected mice (p<0.05 for both compounds). Differences in the bacterial burdens in livers of mice treated with ADEP B315 and *des*-methyl ADEP 4 were not statistically significant. (S1 Dataset).

### Toxicology

As shown in Table 1, ADEP B315 did not induce any significant toxicityat concentrations up to 25 μg/mL in the *in vitro* experiments with human whole blood cultures. As is the case for most antibacterial agents, some cellular toxicity was observed at higher concentrations- 100 μg/mL (p = 0.004 compared to vehicle) and 250 μg/mL (p = 0.03 compared to vehicle).

**Table 1:** *In vitro* toxicity in whole blood cultures.

	Mean number of cells in whole blood culture samples		
Concentration of compound in μg/ml	ADEP B315	Vehicle (matched volume)	p-value for the comparison between ADEP B315 and vehicle (n = 5)
5	71.44	77.3	0.51
10	71.38	74.8	0.45
25	66.98	74.02	0.15
100	30.42	68.08	0.004
250	23.66	51.8	0.03

**Fig 4:**
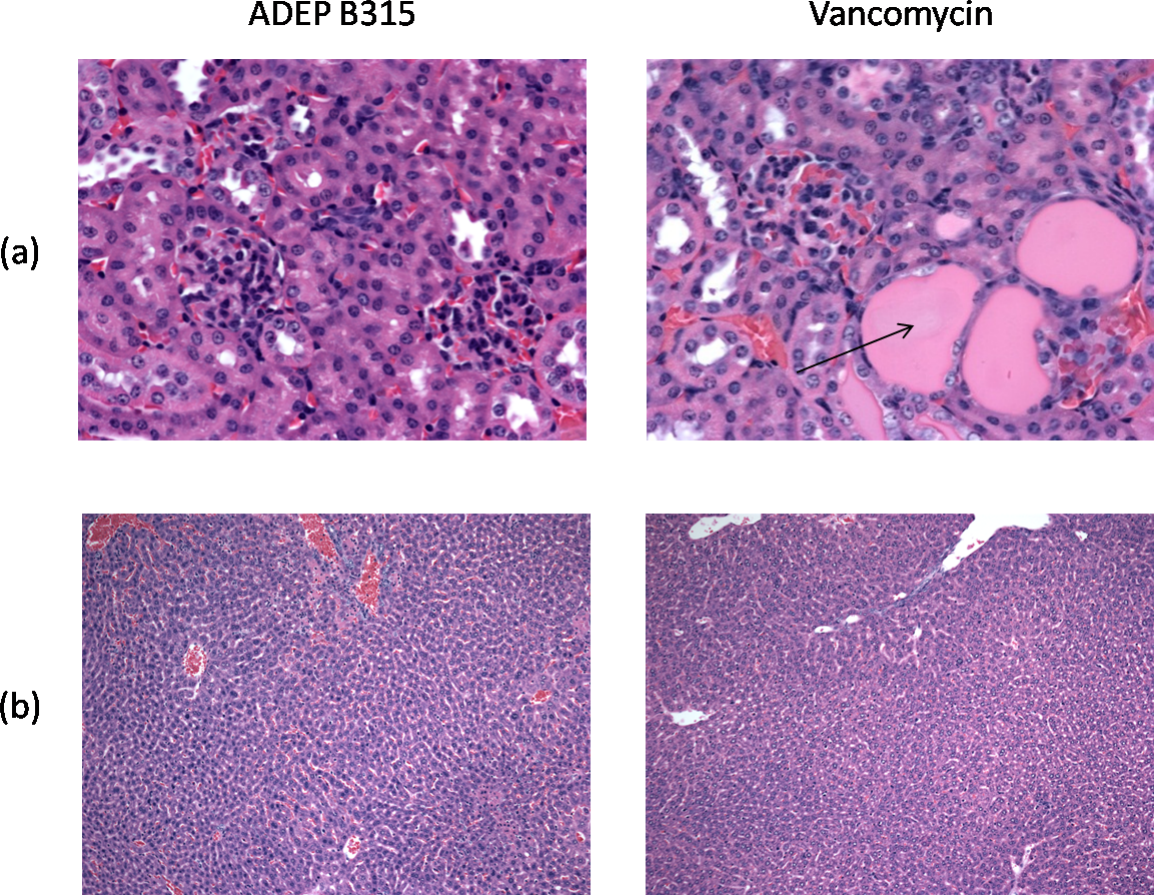
**Microscopic pictures of sections of the kidney (a) and liver (b) of mice treated with 50 mg/kg ADEP B315 or vancomycin, stained with hematoxylin & eosin.** The kidneys of mice treated with vancomycin contained dilated tubules with extensive eosinophilic protein accumulation (arrow) consistent with nephrotoxicity, while normal histology is found in mice treated with ADEP B315.

Photomicrographs of the histological sections of organs from healthy mice treated with 50 mg/kg of either ADEP B315 or vancomycin are shown in Fig 4. ADEP B315 exhibited no significant toxicity on kidney or liver cells and did not affect tissue morphology. In contrast, vancomycin treatment caused vacuolization of the kidney cells and protein accumulation at the same dose, consistent with nephrotoxicity.

## Discussion

Given that the ADEPs have a completely different mechanism of action than all known antibacterial agents, there is optimism that they will be an important tool in a clinician’s arsenal in their fight against organisms that are resistant to other antibacterial compounds [4, 11, 15].The objective of these studies was to assess the *in vivo* efficacy of ADEP B315, a conformationally constrained analog of ADEP 4 that has the most potent *in vitro* activity reported for any member of this new class of antibacterial agents [13] and give some initial information regarding its toxicity. We elected to assess the efficacy of this structurally optimized ADEP in the mouse models of *S*. *aureus* peritonitis in which ADEP 4 was reported to be efficacious [4]. *S*. *aureus* is a well-recognized Gram-positive pathogen that is known to cause a variety of often difficult-to-treat human infections, including skin and soft tissue infections but also invasive infections, such as pneumonia or endocarditis. This pathogen is also notorious for developing antimicrobial resistance thereby severely limiting our antimicrobial options. The mouse model of *S*.*aureus*peritonitis that we selected is often used as an important pre-clinical assay in antibacterial development before moving to tests in larger animals or humans because the murine immune system that is very similar to that of humans [16, 17].

Using the mouse model of peritonitis, we assessed the efficacy of ADEP B315 against methicillin-sensitive and methicillin-resistant strains of *S*. *aureus*. Thestrain of MSSA that we selected is widely used in laboratory experiments [18] and the latter strain is a commonly used clinical isolate [19]. These strains were also selected because they are susceptible to vancomycin, an antibacterial agent that is often the drug of choice for infections caused by multi-drug resistant bacteria [18, 19]. We found that ADEP B315 is effective *in vivo* against a rapidly lethal MSSA infection. Our MSSA assay features rapid lethality of infected mice, with >50% of control mice dying in less than 4 hours after inoculation. Therefore, pre-treatment with the tested compounds 30 minutes before inoculation was deemed necessary to observe a survival benefit. In the MSSA infection model, ADEP B315 was comparable in efficacy to vancomycin,an antibacterial drug that was used as a positive control in the experiments. Interestingly, in our MRSA assay, we found that treatment with ADEP B315 was able to significantly prolong survival of mice infected with the MRSA strain, whereastreatment with vancomycin at the same dose was ineffective. Unlike the experiments with MSSA, pre-treatment was not necessary in the experiments with MRSA. Importantly, we note that our MRSA survival results for the control and the vancomycin group of mice are similar to those that have been previously reported by other groups for the same model of infection [20]. We also find that ADEP B315 is markedly more efficacious than a *des*-methyl analog of ADEP 4, which has MICs that are only two-fold higher than ADEP 4 in *in vitro* assays [4, 11].Furthermore, the antibacterial potency of the optimized ADEP was also reflected in the bacterial burden of infected mice. Specifically, mice treated with ADEP B315 had significantly lowerbacterial cfus/gram of liver and spleen tissue than those treated with the vehicle. Importantly, ADEP B315 exhibited no kidney or liver toxicity in mice at high doses (50mg/kg), and no significant toxicity against human whole blood cells *in vitro* at doses up to 25 μg/mL.

Regarding the limitations of our study, firstly, the rapid time-kill kinetics of our mice experiments and especially the MSSA infection model, requiring pre-treatment with the compounds to achieve meaningful clinical efficacy does not allow for direct comparisons between the MRSA and MSSA infection models. In addition, we were unable to obtain ADEP 4 for our experiments and used its des-methyl analog instead, therefore precluding our ability to make definitive inference about the relative efficacy of ADEP B315 and ADEP 4. Moreover, although we did not find significant *in vivo* toxicity of ADEP B315 at the doses used for a clinical effect in our models,detailed pharmacokinetic/pharmacodynamic experiments about ADEP B315 are required for more exhaustive toxicology evaluation of the compound. Finally, we should note that although we found that the novel ADEP compounds are stable in the vehicle for several days after mixing, it is known that the lactone bond in cyclic depsipeptides is prone to hydrolysis by esterases and this instability often leads to inactivation of the antibacterial properties of the compounds as is the case with daptomycin [21]. Detailed report of the stability of the novel ADEP compound, along with its protein binding and metabolism goes beyond the purpose of our study but remains an important question that should be addressed in future experiments.

In summary, our study indicates that ADEP B315, a structurally optimized cyclic acyldepsipeptide with potent antibacterial activity *in vitro*, is also highly active *in vivo*. We demonstrate that it isa safe and efficacious antibacterial agent for the treatment of lethal infections in animals caused by both sensitive and resistant *S*. *aureus*. Further, ADEP B315 was found to be superior to vancomycin in treating mice infected with MRSA. Future studies will further define the safety profile this novel antibacterial agent and its capacity to treat infections caused by multidrug resistant pathogens in humans.

## Supporting Information

S1 Dataset Minimal dataset for the mice experiments. (XLSX)
